# Editorial: Infodemic management in public health crises

**DOI:** 10.3389/fpubh.2024.1536378

**Published:** 2024-12-17

**Authors:** Dilek Aslan, Fatjona Kamberi, Selen Yegenoglu

**Affiliations:** ^1^Department of Public Health, Faculty of Medicine, Hacettepe University, Ankara, Türkiye; ^2^Scientific Research Centre for Public Health, University of Vlore “Ismail Qemali”, Vlorë, Albania; ^3^Department of Pharmacy Management, Faculty of Pharmacy, Hacettepe University, Ankara, Türkiye

**Keywords:** infodemic, infodemic management, public health, crises, evidence

*Infodemic*, one of the major challenges of the 21st century, is defined as the information overload in digital and/or physical environments, including misinformation, disinformation, unchecked information, information voids, conspiracy theories, etc. *Infodemic* causes risk-taking behaviors that are harmful for health (1). *Infodemic* is not a new phenomenon, as human history has examples with different topics in different time periods (2, 3). On the other hand, public health crises make the communities more vulnerable to *infodemic* and this makes the situation more complex. A recent example has been the Coronavirus-2019 (COVID-19) pandemic. COVID-19 pandemic has shown us how a public health crisis might have crashed the systems of the countries. The world has experienced that it was not only the pandemic itself but also many determinants of health, like social, structural, economical, commercial, and digital, concurrently influenced the course of the pandemic. Thus, the information ecosystem has been one of the major drivers of the burden. *Infodemic* has been a priority to be managed. In this regard, *infodemic management* has been developed as a systematic approach. *Infodemic management* is based on four pillars, including listening to community concerns and questions, facilitating to understand the risk, promoting to understand the advice of the health professionals, maintaining the resilience of the communities, and succeeding to engage and empower them to take intended and positive actions against *infodemic*.

For translating *infodemic management* into future generations, its pillars should be supported with evidence-based science to understand the causes of *infodemic* and to propose sustainable solutions to overcome the problems of today. Such initiatives will be helpful for predicting future risks and taking sustainable, evidence-based measures. At this point, research articles, perspectives, reviews, and opinions might have the potential to contribute to proposing solutions for the current and future crises at the global level. Such evidence is believed to play a crucial role in shaping future perspectives of *infodemic management*. Current evidence will also highlight the need for research gaps and capacity building on the Research Topic. The context of public health and health promotion provides the interdisciplinary feature of *infodemic management*.

**Figure 1:**
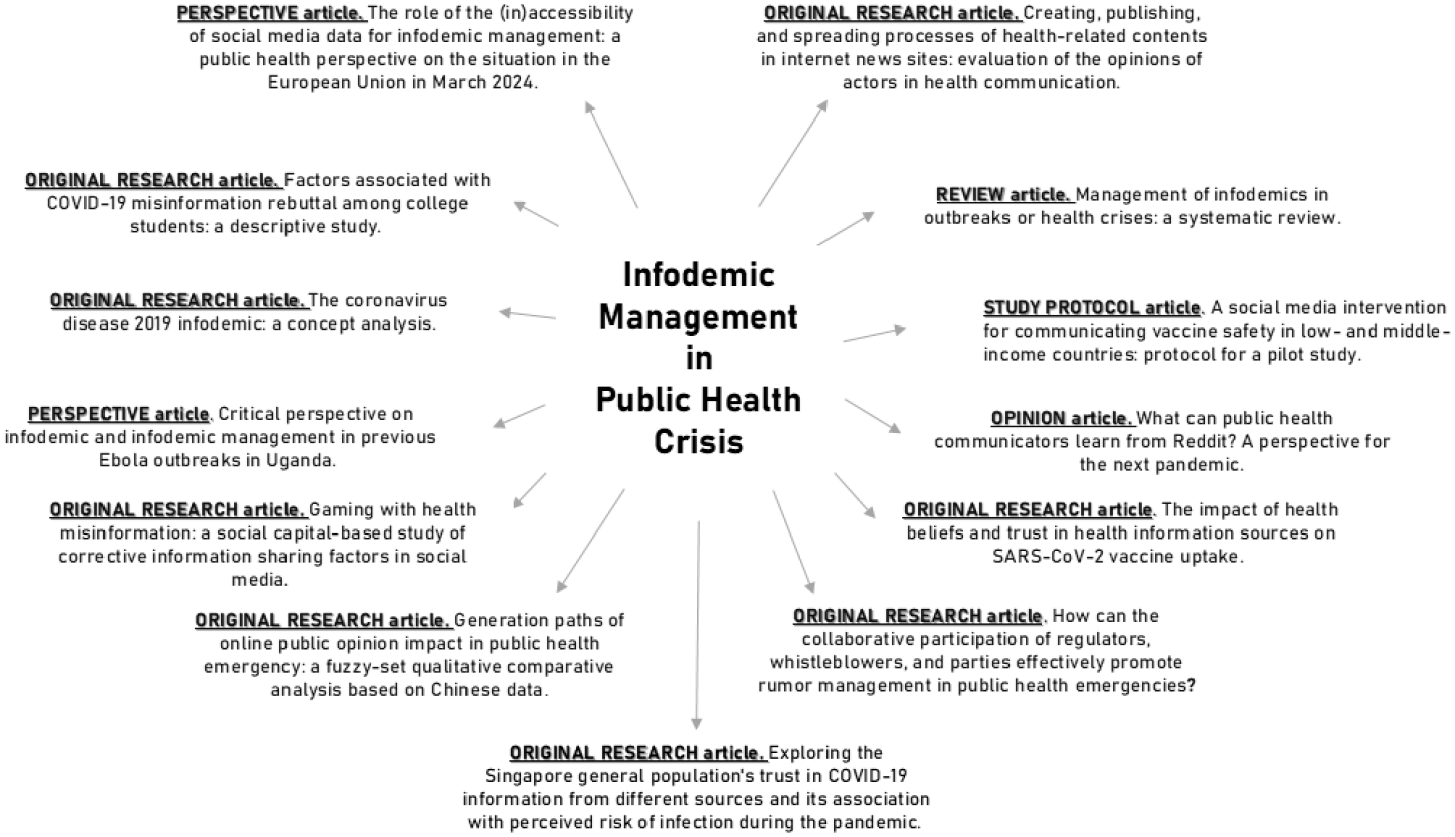
Research Topic collection: *Infodemic management in public health crises*.

Under this Research Topic, different aspects of *infodemic* and *infodemic management* have been tackled from the inter- and transdisciplinary features based on eight original research articles, two perspective articles, one study protocol article, one review, and one opinion article (Figure 1).

Let us briefly emphasize the importance of the articles contributions to the Research Topic. Factors associated with COVID-19 misinformation among college students have been investigated by Shan and Ji from China. Their study emphasized the importance of complexity, dynamics, and differences in online susceptibility of the college students. Devi et al. from Singapore investigated the general population's trust levels in different stakeholders in COVID-19 information from different sources. They showed us that perceived risk of infection is associated with the trust level. Alzahrani from Saudi Arabia studied the impact of health beliefs and trust in health information sources on vaccine uptake. He emphasized the crucial role of targeting health beliefs of the community. Choi from the Republic of Korea performed a concept analysis on the COVID-19 infodemic. The study results might be helpful for the governments and health professionals for building up a policy in order to prevent *infodemic*. Öntaş et al. from Türkiye investigated the internet news sites to assess the opinions of health communication actors. Feng from China did research on the three dimensions of social capital at the theoretical level and provided empirical evidence for specific practices like improving the health literacy of the social media users. *Infodemic* types, including rumors in public health emergencies, were studied by Wang et al. from China. Liu et al. from China shared their qualitative research results. Perspective articles of the Research Topic contributed to widening the view and the scope in infodemic management during public health crises. Ebola outbreak in Uganda and the role of inaccessibility of social media data for *infodemic management* from the European Union widen our vision. The opinion article of the Research Topic gave us different examples of different social media platforms. Such examples are extremely helpful to be prepared for the next pandemic(s). One study protocol article in its pilot study phase written by authors with different backgrounds and institutions in the Research Topic has been a good example of a worldwide network of websites that could be used for effective and good communication. The systematic review article in the content has been a very significant example of how a systematic review can be conducted in the *management* of *infodemic* in health crises.

In the light of the above we feel that more steps should be taken to solve the *infodemic* problem at the global level. We are sure that public health crises' complexity makes everything worse and difficult. In this regard, scientific evidence is recommended to be conducted compatible with the pillars of *infodemic management*. Thus, interdisciplinary and transdisciplinary research teams have to study together to define the challenges and to propose solutions to be prepared for the next public health crises.

Unfortunately, public health crises are today's reality. They are also hot topics of the current century. Therefore, practical, reliable, and objective solutions should meet the needs of the Community. Science and scientific evidence will be the leading tools as they were in the past.

*In conclusion*, the Research Topic on *Infodemic management in public health crises* hopefully will tackle the recent needs and open a new vision for the readers. It provides valuable insights into managing health information during outbreaks like COVID-19. It emphasizes the importance of addressing infodemic, to better control disease spread. The studies highlight the role of social media platforms in shaping communication strategies for future crises including pandemics. By leveraging social capital, improving access to social media data, and learning from past health crises, the findings offer key strategies for enhancing infodemic management in future public health emergencies.
